# Missing pouches in high‐density mapping of atrial tachyarrhythmia in congenital heart diseases

**DOI:** 10.1002/joa3.12251

**Published:** 2019-10-23

**Authors:** Sit‐Yee Kwok, Tak‐Cheung Yung, Ngai‐Lun Ho, Jo‐Jo Hai, Sabrina Tsao, Hung‐Fat Tse

**Affiliations:** ^1^ Department of Paediatric Cardiology Queen Mary Hospital Hong Kong; ^2^ Cardiology Division Department of Medicine Queen Mary Hospital The University of Hong Kong Hong Kong

**Keywords:** atrial tachycardia, catheter ablation, congenital heart disease, high density, intraatrial reentrant tachycardia

## Abstract

**Background:**

The use of high‐density electroanatomical mapping in the Chinese population for congenital heart disease (CHD) is not well reported.

**Methods:**

Retrospective review of consecutive transcatheter ablation of atrial tachyarrhythmia using high‐density mapping for CHD patients (at least moderate complexity) in the only tertiary congenital heart center in the territory from January 2017 to January 2019 was conducted. Orion mapping catheter in Rhythmia system (Boston Scientific) was used to create activation and voltage maps. Parameters including mechanism of arrhythmia, acute success, and follow‐up data were recorded.

**Results:**

Eight patients were identified (median age 35.5 years) who underwent transcatheter ablation of atrial arrhythmia. More than one reentry circuits of IART were identified in five patients. It took a median of 32.4 minutes with 15,952 (IQR 13,395‐18,530) mapping points per map. Cavo‐annulus isthmus‐dependent mechanism was the predominant reentry mechanism. Acute success with the elimination of all inducible tachycardia was achieved in six patients (75%), and partial success in two patients. There was recurrence of atrial arrhythmia in four patients (50%), in which three patients could be maintained in sinus rhythm with low‐dose antiarrhythmic medication. Targeted substrate ablation was performed in six patients with multiple IART circuits. Critical anatomical pouches were identified in three patients, which were missed in the initial mapping using Orion basket mapping catheter.

**Conclusions:**

High acute success rate of atrial arrhythmia ablation can be achieved using high‐density anatomical mapping in CHD. Substrate ablation was required with multiple IART circuits identified. Vigilance should be sought to identify anatomical pouches.

## Graphical abstract

High acute success rate of atrial arrhythmia ablation can be achieved using high‐density anatomical mapping in CHD. Vigilance should be sought to identify anatomical pouches.

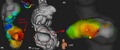

## 1 INTRODUCTION

Transcatheter ablation for atrial tachyarrhythmia in congenital heart disease (CHD) has now been recognized as one of the major treatment options but remains challenging. The challenges include the presence of significantly dilated and/or hypertrophied atrium due to pressure‐ and volume loading, the potential abnormally located or otherwise impaired conduction system, and the arrhythmia substrate altered by fibrosis and/or surgically acquired scars.^1^ Several technical advances including three‐dimensional (3D) image integration, 3D mapping, and remote magnetic navigation, have been employed to facilitate the accurate localization of arrhythmia substrate and improve ablation success.^2^, ^3^, ^4^

A new mapping system (Rhythmia, Boston Scientific) that uses a 64‐mini‐electrode small basket array (IntellaMap Orion, Boston Scientific) enables rapid high‐density mapping in a short time. We aim to report the use of this advanced technology in our Chinese CHD patients.

## 2 METHODS

This is a retrospective review of consecutive transcatheter ablation using high‐density electroanatomical mapping, for atrial tachyarrhythmia of CHD (at least moderate degree of complexity) patients,^5^ from January 2017 to January 2019, in the only tertiary CHD center in the territory. Computed tomography of the heart was performed before the procedure and the scans were reconstructed on the Rhythmia mapping software and merged with the 3D chamber of interest. All antiarrhythmic medication was discontinued for at least five times the elimination half‐life, except one patient with active intraatrial reentrant tachycardia (IART) forgot to stop amiodarone until the day before ablation. The procedures were performed under local anesthetic, sedation, or general anesthesia where appropriate. A 5Fr 4‐pole electrode catheter was placed into the ventricle (retrograde approach in univentricular patients). A 7Fr duodecapolar electrode catheter was placed into the coronary sinus, or in the free wall of the right atrium and it was used for pacing maneuvers and as a timing reference for the high‐density mapping. High‐density electroanatomical mapping was created using Orion mapping catheter in Rhythmia system (Boston Scientific). Before mapping using the Orion catheter, intravenous heparin was used to maintain the activated clotting time between 250 and 350 seconds. Maps and intracardiac signals were acquired predominantly through the Orion, but additional mapping was added if necessary using the ablation catheter. IART was diagnosed as an intraatrial circus movement tachycardia with a constant cycle length inducible by programmed atrial stimulation or burst atrial stimulation. Focal atrial tachycardia (FAT) was defined as tachycardia with centrifugal spread of atrial activation from a focus other than the sinoatrial node. Voltage mapping was performed simultaneously to map scar tissue. During sinus and during tachycardia bipolar signals with filters were used. The activation time at each site was displayed in color relative to the timing reference. Voltage value was recorded at each point and represented in color scale (gray <0.1 mV, purple >0.5 mV). Scar area was defined as tissue with voltage <0.1 mV.

A bidirectional mapping and ablation catheter (IntellaNav, Boston Scientific) was used for ablation after identification of the arrhythmia substrate. For FAT, RF was applied to the earliest atrial activation. For IART, after verification of the critical parts of the reentry circuit, the ablation lesions were created point‐by‐point. For substrate map‐guided ablation, lesions were placed along the entire path between two nonconducting barriers in order to anatomically transect the critical isthmus. Testing for isthmus block was left to the discretion of the treating electrophysiologist and was not required for the definition of success. RF generator power setting was 25‐50 W in the atrium. Successful catheter ablation was defined as the termination of IART/FAT during ablation and failure to reinduce tachycardia at the end of catheter ablation. Partial success was defined as elimination of ≥1 but not all inducible tachycardias. The proof of conduction block along the induced RF line was required as an additional endpoint.

After conclusion of the procedure, patients were monitored in the hospital for at least 24 hours. Any recurring symptoms prompted immediate review and documentation of further arrhythmia was carried out. Major complications were defined as situations requiring additional diagnostic procedures or any specific therapy beyond standard procedural care. Follow‐up data include need for repeat catheter ablation procedures and time to recurrence after the final catheter ablation procedure.

All statistical analyses were performed using SPSS version 24.0 (IBM Somers). Data are presented as mean ± standard deviation (SD) or as median with the respective interquartile range (IQR). Kaplan‐Meier survival analysis was conducted to study freedom from atrial arrhythmia recurrence. P‐values <.05 were considered to be significant.

## 3 RESULTS

A total of eight patients with CHD (median age at ablation 35.5 years, IQR 31.2‐42.1 years, one female) underwent transcatheter ablation of atrial tachyarrhythmia. Diagnosis ranged from repaired tetralogy of Fallot/double outlet right ventricle, congenitally corrected transposition of great arteries, pulmonary atresia with intact ventricular septum postrepair, to atriopulmonary Fontan operation (Table 1). One patient had previously undergone device implantation with an implantable cardioverter‐defibrillator. Two patients had failed previous transcatheter ablation. Four patients presented with ongoing or spontaneous occurring arrhythmia at ablation. In four patients, additional maneuvers like programmed atrial stimulation and/or pharmacologic provocation were required to have sustained atrial tachyarrhythmia for high‐density mapping.

**Table 1:** Patient characteristics and transcatheter ablation summary

Case	Age (years)	Sex	Diagnosis	Tachycardia mechanism	Ablation site	Substrate ablation	Acute success?	EGM	Reference catheter position	AT recurrence
1	36.6	M	Atriopulmonary Fontan (double inlet RV, hypoplastic LV, VA discordance, VSD)	Focal AT	AT focus in right atrial pouch	No	Yes	18,192	Right atrium	Yes
2^a^	41.5	M	ccTGA, severe PS, ASD	Focal AT IART IART JT/AVNRT	Crista terminalis Right posterolateral ablation line Cavo‐annular isthmus ablation NOT ablated	Yes	Partial	17,785 15,745 17,688	Coronary sinus	Yes
3	56.4	M	Repaired TOF	IART Focal AT (non‐sustained)	CTI ablation NOT ablated	No	Yes (confirmed CTI block)	10,882/18,825	Coronary sinus	Yes
4^a^	29.4	F	Atriopulmonary Fontan (double inlet LV, hypoplastic RV)	IART (multiple loops) Focal AT	Lateral RA line creation Septal RA	Yes	Partial	14,379 10,764	Coronary sinus	No
5	34.4	M	Atriopulmonary Fontan (situs inversus, AV discordance, dominant RV, rudimentary LV, double outlet RV, PS)	IART IART	Posterolateral RA line creation cavo‐annular isthmus ablation	Yes	Yes (confirmed isthmus block)	31,306 29,691	Coronary sinus	No
6	31.1	M	Pulmonary atresia, intact ventricular septum s/p pulmonary valvotomy and PVR	IART IART (multiple loops)	CTI ablation posterolateral RA line creation	Yes	Yes (confirmed CTI block)	10,594 14,251	Coronary sinus	No
7	43.7	M	Hypoplastic bipartite RV, ASD s/p ASD closure	IART IART/AT (multiple loops) Focal AT Focal AT	CTI ablation posterolateral RA line creation	Yes	Yes (confirmed CTI block)	50,928 16,158	Coronary sinus	No
8	31.3	M	Dextrocardia, double outlet RV, subaortic VSD, PS s/p surgical repair; myocardial infarction s/p CRTD implantation	IART IART	CTI ablation lateral RA (atriotomy) line creation	Yes	Yes (confirmed CTI block)	10,677 14,233	Right atrium	Yes

Abbreviations: ASD, atrial septal defect; AT, atrial tachycardia; AV discordance, atrioventricular discordance; AVNRT, atrio‐ventricular nodal reentry tachycardia; ccTGA, Congenitally corrected transposition of great arteries; CRTD, Cardiac Resynchronization Therapy Defibrillator; IART, intraatrial reentrant tachycardia; JT, junctional tachycardia; LV, left ventricle; PS, pulmonary stenosis; PVR, pulmonary valve replacement; RV, right ventricle; TOF, Tetralogy of Fallot; VA discordance, ventricular‐arterial discordance; VSD, ventricular septal defect.

a Redo case of transcatheter ablation.

A total of 16 high resolution maps were acquired with the new mini‐basket catheter. Incomplete mapping of IART (defined as less than 90% of TCL) was not included as analysis. This was common due to nonsustained AT, termination into sinus rhythm, or degeneration into different tachycardia. The median collected electrograms per tachycardia was 15,952 (IQR 13,395‐18,350), with a median atrial tachycardia cycle length of 275 milliseconds. A median of two maps per patient was finally completed. The median time to obtain a single map was 32.4 minutes (IQR 15.6‐50.6 minutes). All patients had atrial tachyarrhythmia (FAT‐1; IART‐3; both‐4). More than one reentry circuits of IART were identified in five patients, in which three patients had three or more IART mechanisms. Of the seven patients with IART, all except one patient had cavo‐annulus isthmus‐dependent mechanism as one of the reentry mechanism. For noncavo‐annulus isthmus‐dependent IART, lateral or posterolateral right atrium was the most common site of critical isthmus location. This area corresponds to surgical atriotomy incision in most of the cases.

Acute success with the elimination of all inducible tachycardia was achieved in six patients (75%), and partial success in three patients (25%).

**Figure 1:**
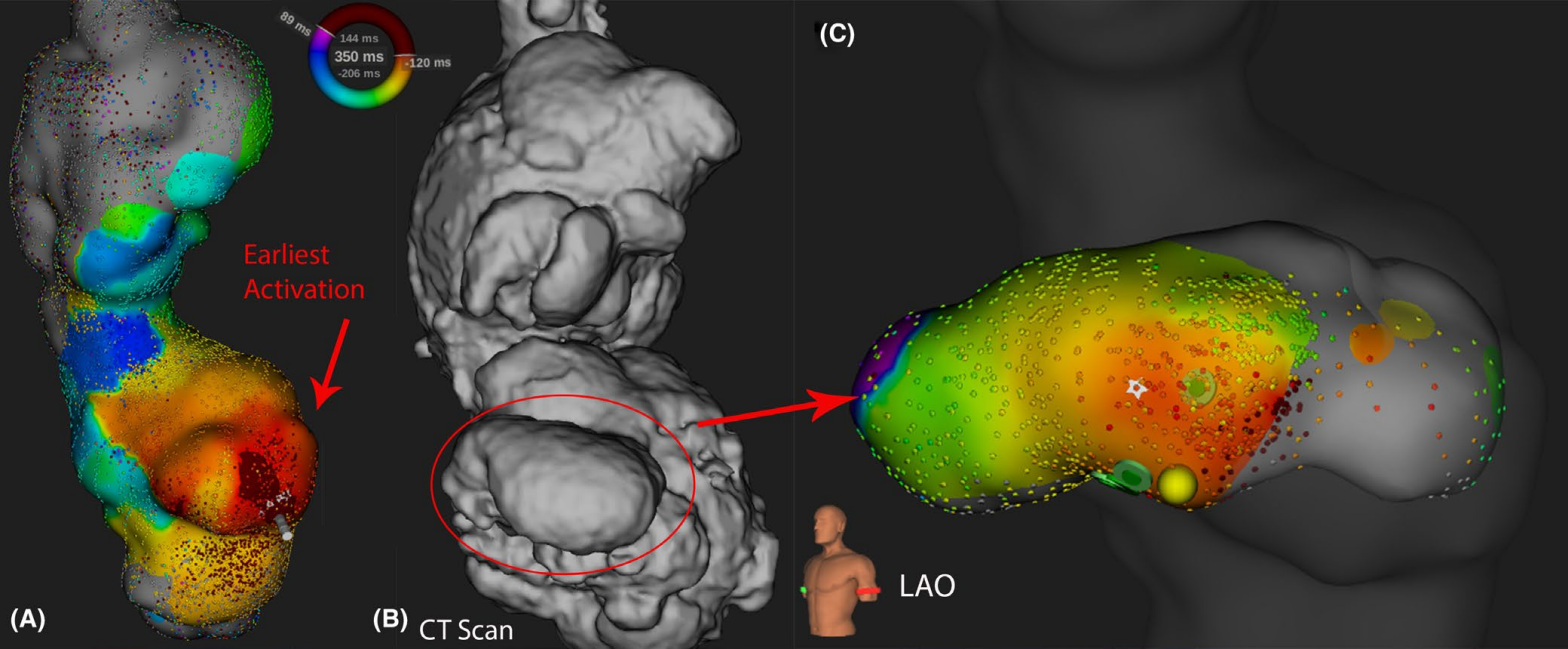
Example of a 36‐year‐old male patient with atriopulmonary Fontan (case 1), with nearly missed anatomical pouch. The origin of FAT was identified within the pouch. (See text for details)

In three patients, anatomical pouches were nearly missed in the initial mapping, which were part of the arrhythmia substrate:

The first patient (Case 1) was a 36‐year‐old male with atriopulmonary Fontan. Initial high‐density mapping suggested the origin of the focal atrial tachycardia was located over anterior right atrium (Figure 1A). However, no good unipolar signals could be identified. With careful comparison with the preprocedural CT, a small pouch was found medial to the earliest activation area (Figure 1B). Deflation of the Orion basket catheter was required to enter the pouch and 4609 electrograms were recorded inside the right atrial pouch (Figure 1C). Radiofrequency ablation was performed at the site with earliest activation, with the termination of atrial tachycardia.

**Figure 2:**
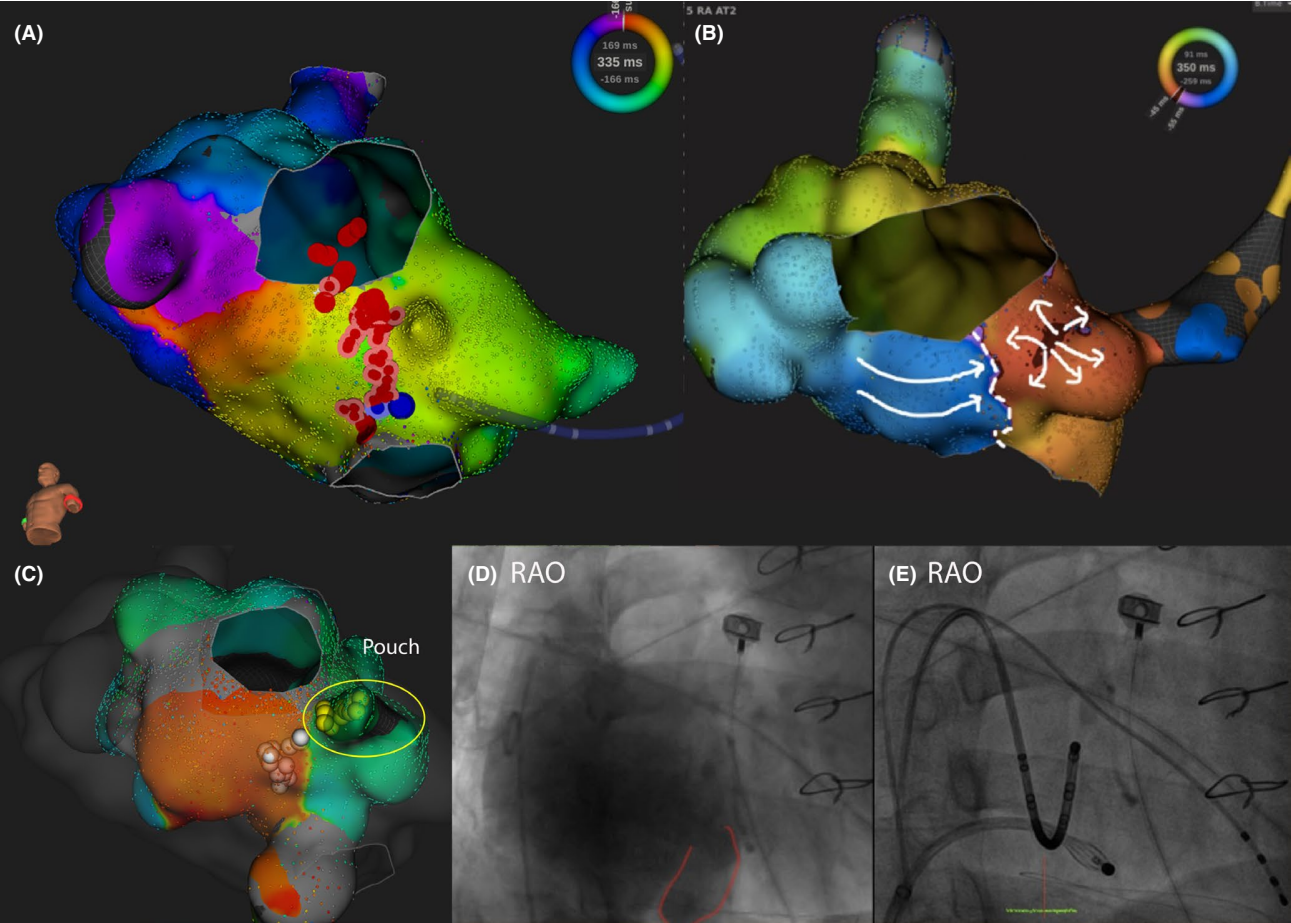
A 31‐year‐old male with pulmonary atresia with intact ventricular septum s/p surgical total repair (case 6). CTI ablation (red dots) was performed (blue spot as termination spot). Incomplete CTI ablation was suggested with breakthrough medial to the line from which the atrial activation propagated. An anatomical pouch was identified and further ablation was performed (yellow dots) inside the pouch. The posterior CTI line was reinforced (pink dots), while the ablation line was extended toward the anatomical pouch (white dots). (See text for details)

Both case 3 and 6 demonstrated similar pouches not identified during the cavo‐annulus isthmus ablation. Case 6 was a 31‐year‐old male with pulmonary atresia with intact ventricular septum s/p surgical total repair, tricuspid ring annuloplasty and pulmonary valve replacement, and previous surgical right‐sided Maze procedure. High‐density mapping suggested anticlockwise cavo‐tricuspid isthmus (CTI)‐dependent atrial flutter (Figure 2A). CTI ablation line was created and atrial flutter terminated upon completion. However, atrial flutter could still be induced. High‐density propagation mapping suggested there was conduction delay over the created CTI ablation line, but there was a site a few millimeters septal to the line from which the atrial activation propagated (Figure 2B and video S1). Angiogram identified an anatomical pouch over the septal inferior aspect (Figure 2D). Further mapping required dedicated deflation of the Orion mapping catheter to enter the pouch, septal to the CTI line (Figure 2E). Further ablation was performed inside the pouch area (Figure 2C), and validation map postablation confirmed complete blockage.

**Figure 3:**
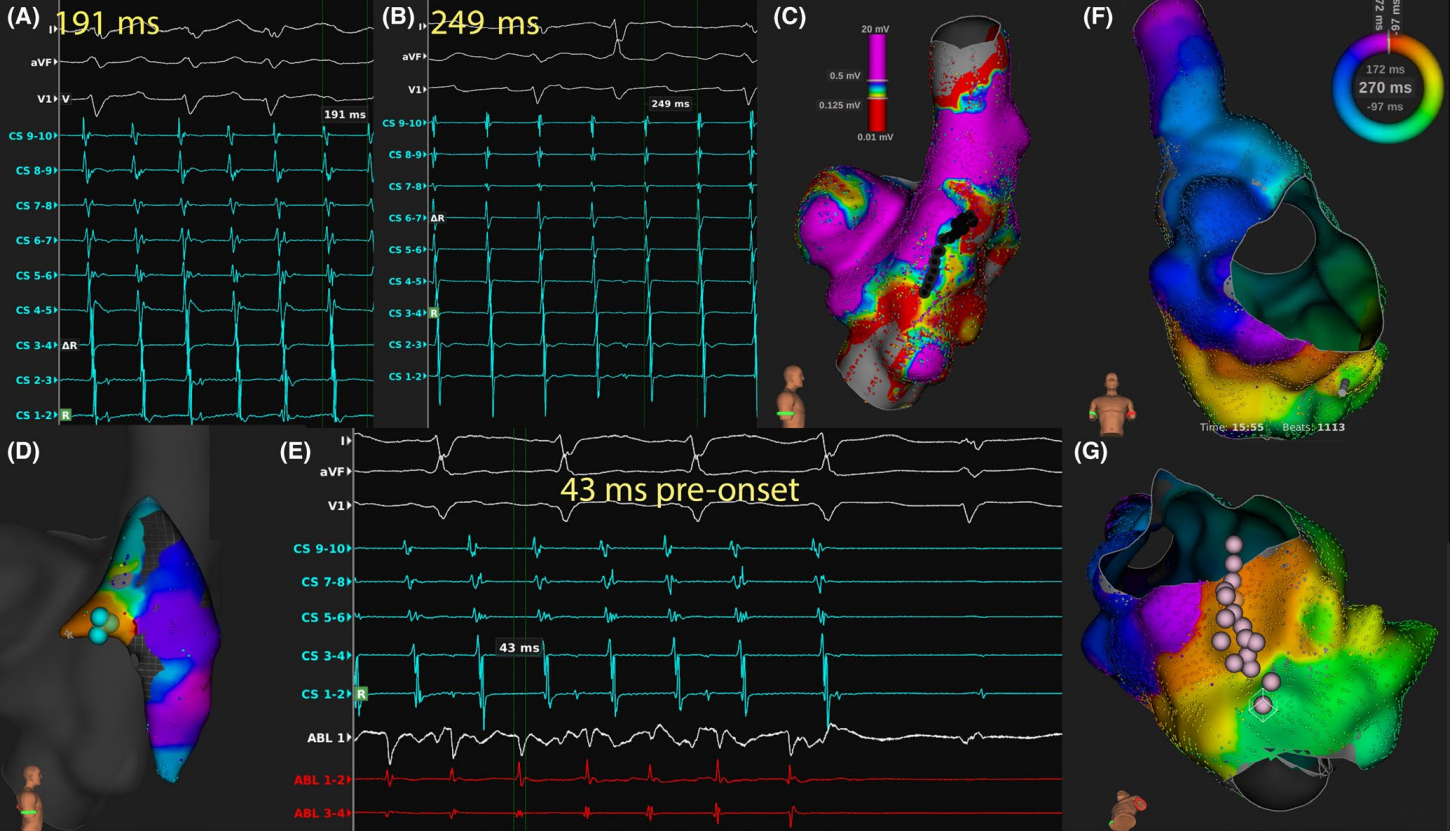
A 41‐year‐old male with congenital corrected transposition of great arteries. (case 2) A and B, Electrophysiological study identified multiple atrial tachycardia with different cycle lengths. C, No single mechanism of atrial tachycardia could be identified. Voltage mapping identified low‐voltage area (red: <0.125 mV; purple >0.5 mV). RF ablation line (black dots) was created to connect scar and low‐voltage region, after which the AT changed. D and E, Focal atrial tachycardia. Earliest atrial activation was identified over the crista terminalis, with local atrial activation 43 ms ahead of the onset of P wave. RF ablation at this region successfully terminated the tachycardia. F, High‐density mapping of another AT suggested clockwise cavo‐annular‐dependent intraatrial reentry tachycardia. G, RF ablation line was created (pink dots) and IART was terminated

**Figure 4:**
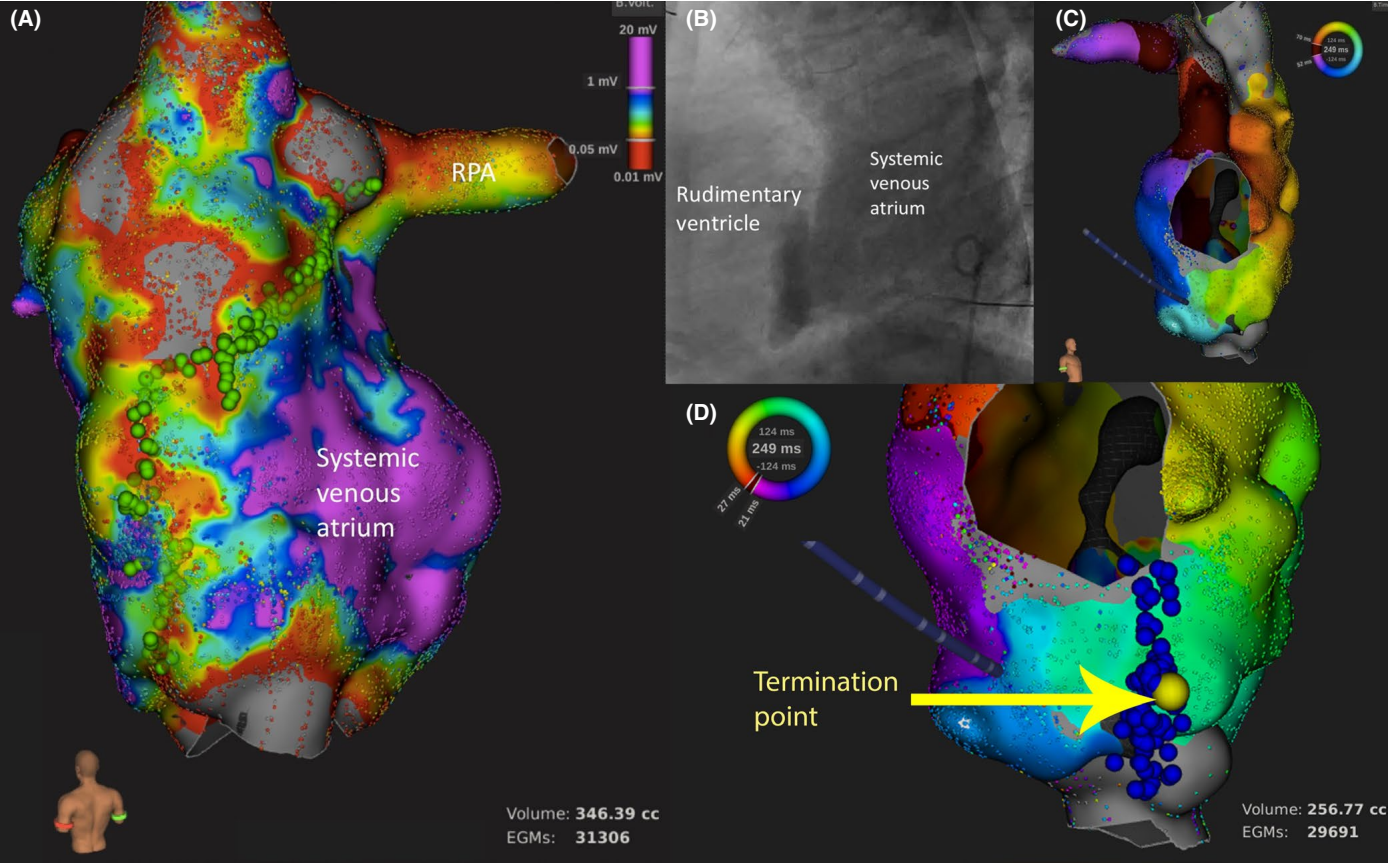
A 34‐year‐old male with situs inversus, dominant right ventricle, rudimentary left ventricular, and pulmonary stenosis s/p atriopulmonary Fontan (case 5). A, Voltage mapping guided the subsequent substrate modification ablation. RF ablation line (green dots) was created to connect the low‐voltage areas from the right pulmonary artery to the inferior vena cava. B, Angiogram (lateral view) demonstrated the systemic venous atrium communicated with the small rudimentary ventricle at the front of the atrium. C, High‐density mapping of systemic venous atrium identified the anticlockwise intraatrial reentrant tachycardia (IART), which was cavo‐annulus isthmus dependent, around the rudimentary ventricle. D, RF ablation (blue dots) successfully terminated the IART

**Figure 5:**
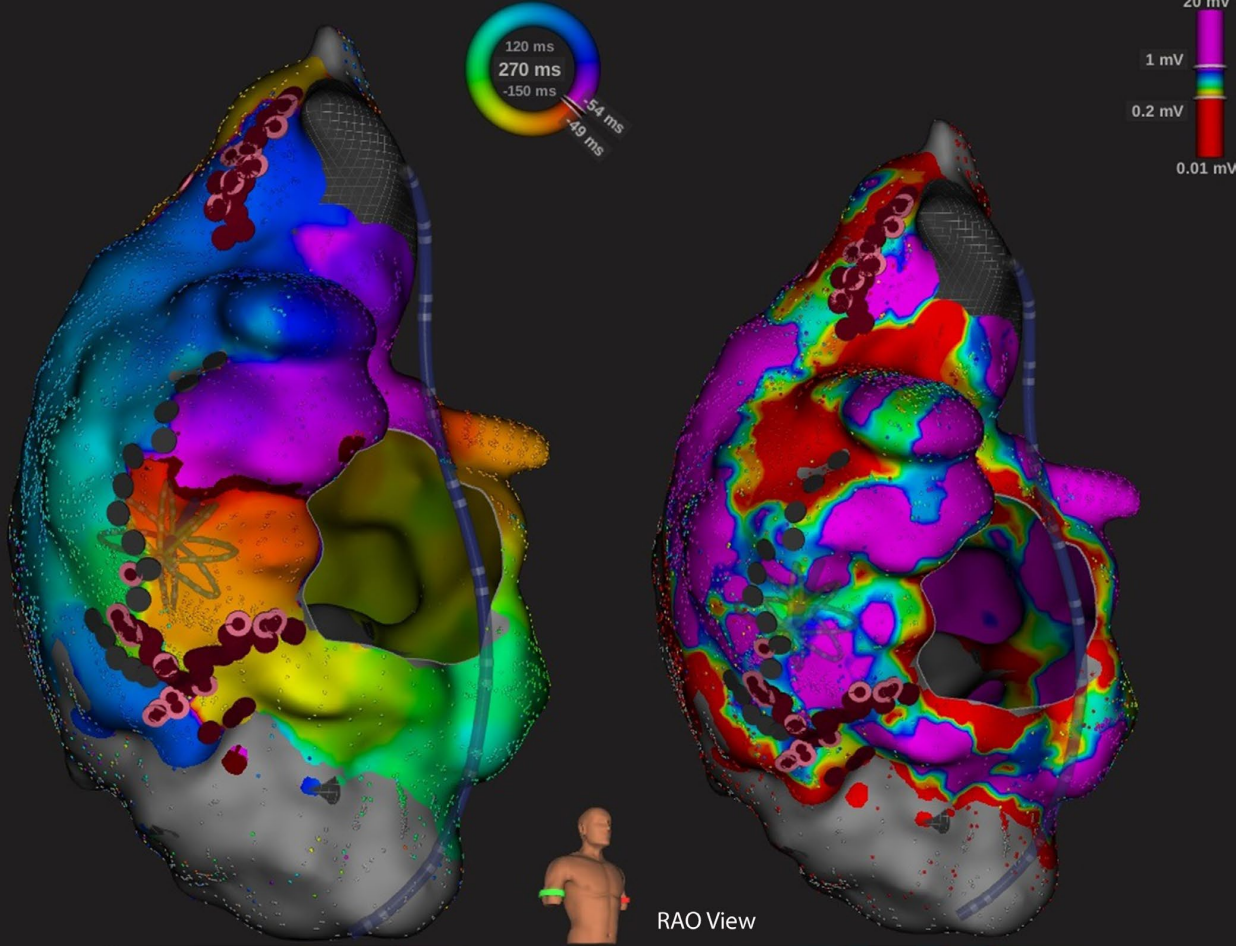
A 31‐year‐old male with double outlet right ventricle, subaortic ventricular septal defect, and pulmonary stenosis posttotal surgical repair (case 8). High‐density activation mapping (left) suggested clockwise IART around a line of block over the right lateral atrium (black dots). This represented previous atriotomy scar. With the aid of the voltage mapping (right), RF substrate ablation was performed to create lines of block and connected the low‐voltage areas: from the superior vena cava to the right atriotomy scar; from the atriotomy scar to the inferior vena cava; and from the tricuspid annulus to the lateral line of block

Voltage mapping was performed in six of eight patients, in which more than one atrial tachyarrhythmia mechanisms were identified. Further substrate ablation was guided by the information obtained from voltage mapping (Figures 3, 4, 5). Procedure duration amounted to a median 463.5 minutes (IQR 446.3‐488.3 minutes). Median fluoroscopy duration was 86.0 minutes (IQR 67.6‐114.7 minutes).

The recurrence rate was 50% (4/8) during median follow‐up time of 14.6 months (IQR 7.3‐17.3 months). Arrhythmia recurrence was observed in four cases (case 1‐3, 8) in which rate control was chosen for one patient. Case 2 underwent DC cardioversion again and sinus rhythm could be maintained with low‐dose sotalol. In case 3, IART was pharmacologically cardioverted with escalating dose of sotalol (lower than the dose preablation), without further breakthrough atrial tachyarrhythmia on maintenance antiarrhythmic medication. Case 8 (with history of atrial fibrillation) had increase in asymptomatic atrial tachyarrhythmia episodes recorded by his CRT‐D at 7 months postablation. Low‐dose sotalol was used and no more atrial tachyarrhythmia thereafter. All patients reported improved symptoms and had less hospitalization due to tachyarrhythmia postablation.

At recent follow‐up (median of 14.6 months), seven of eight patients (87.5%) were in sinus rhythm. No complications in the form of cardiac perforation, pericardial effusion, or tamponade were observed in our study. No mortality was encountered either during the procedure or at recent follow‐up. The atrial tachyarrhythmia recurrence–free survival rates in 1, 4, and 12 months were 87.5%, 62.5%, and 50.0%, respectively.

## 4 DISCUSSION

Over the past decades, 3D electroanatomical mapping systems have been useful for identifying the reentrant circuits and localizing arrhythmogenic channels in macro reentrant tachycardias. It acquired each electrogram by a point‐by‐point method and usually required manual reannotation. A new mapping system (Rhythmia, Boston Scientific) using a small basket array with 64 mini electrodes (IntellaMap Orion, Boston Scientific) has been developed and has enabled rapid high‐density mapping in a short time.^6^, ^7^, ^8^

Patients with congenital heart lesions represent a challenging group with unique cardiac anatomy and atypical arrhythmia substrates. Surgical repair of CHD may affect the anatomy. The hemodynamic changes and surgical palliation predispose CHD patients to having multiple arrhythmia mechanisms. In CHD population, areas of slow conduction and conduction block are common because of surgical scars, surgical patches, and fibrosis. High‐density mapping of arrhythmia mechanism and substrate can be employed in this challenging patient cohort. The initial experience has been recently described by Ernst et al^9^ in Europe, but has not been well reported in Asia‐Pacific region.

The majority of arrhythmia substrates in CHD patients is reentrant in nature and frequently caused by the preceding surgical interventions, which result in scar tissue (eg, atriotomy scar) that can create protected channels of conduction that can serve as critical isthmuses in IART. In addition, this could also be attributed by significant chamber dilatation. Our cohort demonstrated the comparable of atrial tachyarrhythmia mechanisms in CHD, with predominant IART through the cavo‐annular (cavo‐tricuspid) isthmus. In cases of non‐CTI‐related IART, the most frequent location of IART isthmus was the lateral or posterolateral wall of the venous atria.^10^, ^11^, ^12^ Recurrent circuits around caval orifices, crista terminalis, or the atrial appendage are also observed, but less frequently.^13^ We also demonstrated that multiple IARTs can be induced in a single patient, each requiring individual mapping and ablation.

The anatomical pouches were nearly missed in the initial mapping in our three patients. Complex, large chambers seen in CHD pose a challenge to both catheter manipulation and the construction of precise anatomical and electrical maps. Giant, hypertrophied atria are inherent among Fontan patients and often hamper adequate catheter reach. The absence of detailed electroanatomical mapping arising from missed areas of mapping increases the risk of misdiagnosis and may prevent the identification of the optimal ablation site.^13^ These pouches should be carefully looked for as they may form part of the critical isthmus, or hinder the contact of the ablation catheter in creating effective lesions. The initial experience by Ernst et al suggested the acquired 3D map was smaller than the 3D reconstructed chamber anatomy by preprocedural imaging, in half of their procedures.^9^ The bulky basket mapping catheter may hinder entry into these small pouches of atrial tissues, which can be the critical area of anatomical isthmus. Intraprocedural angiograms and adequate preprocedural imaging help the operators to ensure the entire chamber has been mapped, which is also our current practice after gaining the above‐mentioned experience. Sometimes, conventional ablation catheter may also be utilized to negotiate through the neck of the pouch, to complete the pouch mapping.

In the study of Roca‐Luque et al, a cut‐off voltage of 0.5 mV could identify 95.4% of the substrates in non‐CTI‐related IART.^12^ Unexcitable sites can be displayed together with the caval veins, valve annuli, and lines of double potentials. Double potentials may be located between the caval veins and the posterolateral systemic venous atrium consistent with the crista terminalis but may also indicate atriotomy and suture lines. After delineation of all boundaries that create intervening isthmus potentially related to atrial tachyarrhythmia, these isthmuses can be targeted by a liner RF ablation lesion connecting the unexcitable boundaries during sinus rhythm, like in our cases.^14^ Empiric cavo‐annulus and intercaval ablation may be particularly useful in patients with multiple tachycardias, irregular tachycardias or where the tachycardia is noninducible or not hemodynamically tolerated.^15^ This could have contributed to the high acute success rate in our cohort, with the identification of these isthmuses using substrate mapping.

The advantage of using the Rhythmia system is the speedy acquisition of multiple mapping points with the mini basket and its small electrodes. In the study by Rottner et al, Rhythmia‐guided ablation approach, when compared to Carto‐guided ablation, was observed to enable multiple times of mapping points to be collected.^16^ In the study by Anter et al, Rhythmia‐guided ablation demonstrated successful reattempt in ablation, in previous Carto‐ or NavX‐guided failure. The inadequate mapping with Carto‐ or NavX‐guided ablation was attributed to the presence of scar with highly fractionated electrograms, precluding accurate time annotation, and frequent change in the tachycardia. Electrogram duration in low‐voltage areas was found to be shorter with the basket catheter than with the PentaRay catheter.^17^ This suggested the sampling was from smaller tissue size, consistent with the basket's smaller electrodes and close interelectrode spacing. In the area of low voltage, border zone, and scar tissue with complex electrograms, acquisition of multiple points can reduce the potential error of too few data points with erroneous activation times. The fast acquisition and high mapping resolution enables the huge atrium in our CHD to be mapped in a short time, using the Orion basket catheter.

For the disadvantages, the basket mapping catheter was bulky and less steerable when compared to conventional mapping catheter. This posed extra challenges in our cohort as our mapped atria are often huge with abnormal shapes. As mentioned, some anatomical region could have been missed during initial mapping, especially small pouches. The pouches may also hinder the contact of the ablation catheter in creating effective lesions. Differentiation between scar and noncontact is difficult to determine. As illustrated, CHD patients had large atria with lots of scarring and fibrotic area. Operators needed to overcome a learning curve to determine whether the map was complete, with the help of preprocedural and intraoperative imaging. With much thicker myocardium in the CHD patients, it hindered a smooth achievement of ablation line. 40‐50 W of energy was required to complete the CTI ablation line for some of our patients. Technology to monitor whether a transmural lesion has been made (eg, contact force) could greatly help but this was not available in Rhythmia mapping system.

The follow‐up period was relatively short for our cohort. However, as 75% of all recurrence occurred within the first year for previously published cohort,^18^ we believe presenting early recurrence could adequately reflect the effectiveness of our atrial tachyarrhythmia ablation in CHD. We encountered a higher recurrence of atrial tachyarrhythmia for our first few cases, and this is expected as CHD ablation and the use of the system required accumulation of experience. In fact, the AT recurrence rate (50%) is comparable to other similar CHD cohort,^14^, ^19^ in which usually more than one ablation procedures are required. Recently, clinical arrhythmia severity score was used to assess the efficacy of transcatheter ablation in CHD cohort, instead of recurrence rate.^20^ All our patients with recurrence had less severe symptoms postablation, and majority of them could be maintained in sinus rhythm with low‐dose antiarrhythmic medication.

The median fluoroscopy time was relatively long in our cohort, due to our learning curve in utilizing the high‐density mapping system nonfluoroscopically. Aside from CT image, it was our practice to perform fluoroscopic angiograms to define the atrial border. Therefore, extra fluoroscopic guidance was used to ensure complete mapping of the large atrium, to ensure the Orion basket catheter could reach the border. Difficulty to locate hidden anatomical pouches in our cohort also contributed to the extra fluoroscopic use.

## 5 STUDY LIMITATIONS

This was a retrospective study with heterogeneity and is subject to the consequent limitations. The relatively short follow‐up duration also hindered long‐term outcome to be analyzed with our study.

## 6 CONCLUSION

The initial experience of using high‐density mapping in transcatheter ablation of atrial tachyarrhythmia is presented, in a cohort of Chinese CHD patients with at least moderate in complexity. High acute success rate was achieved. Vigilance should be sought to identify anatomical pouches using high‐density mapping in this special cohort of patients.

## CONFLICT OF INTEREST

Authors declare no conflict of interests for this article.

## AUTHOR CONTRIBUTION

SY Kwok was involved in concept/design, data collection, data analysis/interpretation, and drafting article. TC Yung and HF Tse were involved in critical revision of article, approval of article. NL Ho was involved in data analysis/interpretation, statistics. JJ Hai and S Tsao were involved in critical revision of article. 

## ETHICAL APPROVAL

This study received ethics approval from the Institutional Review Board of the University of Hong Kong/Hospital Authority Hong Kong Western Cluster.
